# A standardized motor imagery introduction program (MIIP) for neuro-rehabilitation: development and evaluation

**DOI:** 10.3389/fnhum.2013.00477

**Published:** 2013-08-22

**Authors:** C. Wondrusch, C. Schuster-Amft

**Affiliations:** ^1^School of Health Professions, Institute for Physiotherapy, Zurich University of Applied SciencesWinterthur, Switzerland; ^2^Research Department, Reha RheinfeldenRheinfelden, Switzerland; ^3^Department of Engineering and Information Technology, Institute for Rehabilitation and Performance Technology, Bern University of Applied SciencesBurgdorf, Switzerland

**Keywords:** motor imagery introduction program (MIIP), CNS lesion, sensorimotor impairments, mental practice, stroke, multiple sclerosis, Parkinson's disease

## Abstract

**Background:** For patients with central nervous system (CNS) lesions and sensorimotor impairments a solid motor imagery (MI) introduction is crucial to understand and use MI to improve motor performance. The study's aim was to develop and evaluate a standardized MI group introduction program (MIIP) for patients after stroke, multiple sclerosis (MS), Parkinson's disease (PD), and traumatic brain injury (TBI).

**Methods:** Phase 1: Based on literature a MIIP was developed comprising MI theory (definition, type, mode, perspective, planning) and MI practice (performance, control). Phase 2: Development of a 27-item self-administered MIIP evaluation questionnaire, assessing MI knowledge self-evaluation of the ability to perform MI and patient satisfaction with the MIIP. Phase 3: Evaluation of MIIP and MI questionnaire by 2 independent MI experts based on predefined criteria and 2 patients using semi-structured interviews. Phase 4: Case series with a pre-post design to evaluate MIIP (3 × 30 min) using the MI questionnaire, Imaprax, Kinaesthetic and Visual Imagery Questionnaire, and Mental Chronometry. The paired *t*-test and the Wilcoxon signed-rank test were used to determine significant changes.

**Results:** Data of eleven patients were analysed (5 females; age 62.3 ± 14.1 years). Declarative MI knowledge improved significantly from 5.4 ± 2.2 to 8.8 ± 2.9 (*p* = 0.010). Patients demonstrated good satisfaction with MIIP (mean satisfaction score: 83.2 ± 11.4%). MI ability remained on a high level but showed no significant change, except a significant decrease in the Kinaesthetic and Visual Imagery Questionnaire score.

**Conclusion:** The presented MIIP seems to be valid and feasible for patients with CNS lesions and sensorimotor impairments resulting in improved MI knowledge. MIIP sessions can be held in groups of four or less. MI ability and Mental Chronometry remained unchanged after 3 training sessions.

## Introduction

Motor imagery (MI) is defined “as a dynamic state, during which the representation of a given motor act is internally rehearsed within working memory without any overt motor output” (Decety and Grezes, 1999). It is assumed that action planning, action preparation, action simulation, and action observation share similar neuronal substrates (Decety and Grezes, 1999).

MI as a technique to improve motor performance and function has evolved in sports psychology (Start, 1964), where a positive effect of MI training on motor performance had been confirmed (Casby and Moran, 1998; Smith et al., 2001; Guillot et al., 2010). More than 20 years ago, MI as a therapeutic concept has been implemented into neurorehabilitation for patients with sensorimotor impairments. The idea was to have an additional instrument besides the classical therapies to re-establish motor function (Warner and McNeill, 1988). The advantage for patients has been the opportunity to train affected body parts already at an early stage of rehabilitation, when physical movement was not yet possible. As an additional advantage patients have been able to train safely in absence of a therapist, and to fill spare time in the clinical routine with an effective intervention.

Since the beginning of this millennium, a growing body of research has been conducted to test the efficacy of MI interventions in neurorehabilitation (Barclay-Goddard et al., 2011; Schuster et al., 2011). So far, results of this research have been ambiguous (Ietswaart et al., 2011; Braun et al., 2012; Schuster et al., 2012a), likely due the following reasons: the number of patients included in the majority of studies has been too small to draw solid conclusions, and the heterogeneity between patient characteristics and intervention designs was too large to allow for meaningful comparisons. Furthermore, it is difficult to assess MI objectively due to its “concealed nature” (Guillot and Collet, 2005; Malouin et al., 2008a) and comparison of study results is hampered due to different MI ability assessment tools used (Malouin et al., 2008b; Schuster et al., 2010).

There is consensus, that MI interventions are cognitively complex and challenging (Braun et al., 2008; Bovend'eerdt et al., 2010; Ietswaart et al., 2011; Schuster et al., 2012b). Different frameworks for clinical implementation and practice have been published (Braun et al., 2008; Bovend'eerdt et al., 2010). Furthermore, a single MI training session can vary in different elements, such a position, location, and instruction type (Schuster et al., 2011). The twenty different MI training session elements, described in the literature (Schuster et al., 2011), were developed based on the PETTLEP approach published by Holmes (2001). Some of these elements are highly abstract and require a certain level of cognitive ability to be understood by patients or study participants. For example, MI can be rehearsed in different sensory modalities, such as kinesthetic or visual (Schuster et al., 2011). Furthermore, the patient can take an internal and external perspective (Schuster et al., 2011). Thus, without a clear and standardized introduction or familiarization, MI can be interpreted and practiced in different ways by researchers, study participants, or patients. This could jeopardize the validity of study results and the outcome of therapeutic interventions.

Despite the awareness of the complexity of MI (Heremans et al., 2012; Madan and Singhal, 2012), so far little attention has been paid to the introduction and familiarization process of patients or study participants to the concept of MI prior to a MI intervention. Only 19 of 133 studies included in the literature review about best practice in MI by Schuster et al. mentioned an introduction or familiarization element as part of their MI intervention (Schuster et al., 2011). None of these studies examined the introduction or familiarization as an independent MI training session element. In the absence of a standardized introduction or familiarization session prior to an MI intervention, it could be hypothesized that patients and study participants lack important information that would help them to understand the complexity of MI. This may lead to decreased compliance and to a feeling of excessive demands (Bovend'eerdt et al., 2010; Schuster et al., 2012b). Therefore, it is essential that patients or study participants are carefully introduced to the concept of MI before they are tested or start with MI training programs. To improve MI understanding and the basic MI performance skills declarative and procedural knowledge have to be transferred (Annett, 1996). Declarative knowledge involves “knowing the rule,” whereas procedural knowledge focuses on “applying the rule” (Nickols, 2000). This knowledge might enable patients or study participants to complete MI assessment tests based on reliable knowledge and to start MI training. A solid basis that allows generating comparable data is equally important for clinical practice as for research interventions. In other fields such as low back pain and endodontic, standardized programs for knowledge transfer have been developed and evaluated and have shown to be effective for knowledge increase (Meng et al., 2009; Sorrell et al., 2009; Foltran et al., 2012).

Therefore the aim of this study was the development and the evaluation of a MI introduction program (MIIP) to familiarize patients with sensorimotor impairments due to central nervous system (CNS) lesion with the concept of MI, to transfer important knowledge and therefore, to improve the understanding of the MI concept, to teach basic MI skills, and to increase the self-perception of the skill to perform MI. Our hypothesis was that MIIP would increase patient knowledge about MI, improve self-perception of the skill to perform MI and result in a good overall satisfaction with MI. Furthermore, it was of interest whether such a pre-training program would change MI ability.

## Methods

**Figure 1:**
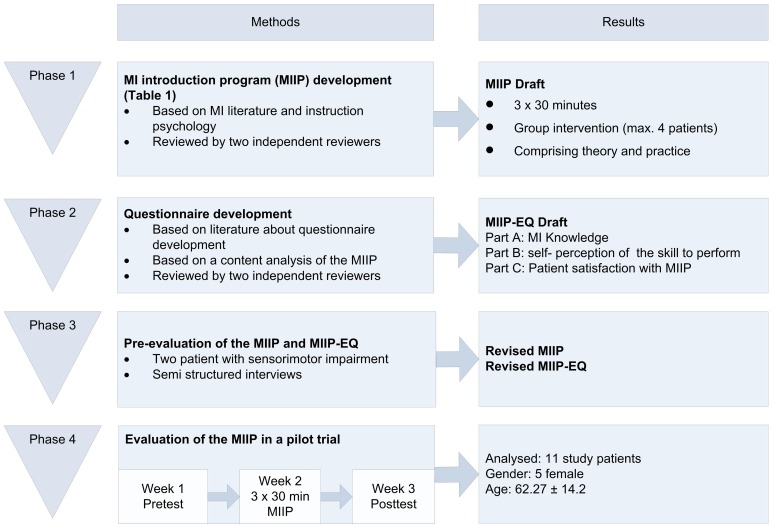
**Study phases**.

The project was divided into four phases: (1) MIIP development, (2) MIIP questionnaire development, (3) Pre-evaluation of the MIIP and the MIIP questionnaire, and (4) Evaluation of the MIIP in a patient pilot trial. An overview of the complete study process is shown in Figure 1. The study was approved by the local ethics committee (Ethikkommission Kanton Aargau Switzerland, Reference number: 2012/050) and was conducted in accordance with the Declaration of Helsinki and Good Clinical Practice guidelines. The study was conducted in a neurorehabilitation center in Northwestern Switzerland and study patients were recruited from the clinic internal database according to their diagnosis.

### Phase 1: development of the motor imagery introduction program (MIIP)

The objective of this phase was the development of a standardized MIIP in German to improve declarative and procedural knowledge about MI in patients with CNS lesions and sensorimotor impairments. To reach this aim, two main issues were regarded as important. First, the content of the program had to be based on published literature and on current best MI practice. Secondly, the collected information had to be presented in a form appropriate for patients with CNS lesions and sensorimotor impairments with the intention to support the knowledge transfer process.

To find out more about current introduction practice, a content analysis and data extraction regarding the details of the MI training session element familiarization was performed for all 19 studies in the review by Schuster et al. (2011) that mentioned a familiarization or introduction session prior to the investigated MI intervention (Clark, 1960; Van Gyn et al., 1990; Etnier and Landers, 1996; Shambrook, 1996; Casby and Moran, 1998; Smith et al., 2001; Dickstein et al., 2004; Kornspan et al., 2004; Liu et al., 2004a; Reiser, 2005; Sidaway and Trzaska, 2005; Dunsky et al., 2006; Yoo and Chung, 2006; Immenroth et al., 2007; Braun et al., 2008; Bovend'eerdt et al., 2009, 2010; Malouin et al., 2009; Hemayattalab and Movahedi, 2010). Subsequently, the corresponding authors of these 19 studies were contacted and asked to provide detailed information about their introduction or familiarization protocol, respectively. Nine authors responded (Sonnenschein, 1990; Casby and Moran, 1998; Dickstein et al., 2004; Liu et al., 2004b; Sidaway and Trzaska, 2005; Dunsky et al., 2006; Braun et al., 2008; Bovend'eerdt et al., 2010; Hemayattalab and Movahedi, 2010) and three of them provided additional information (Casby and Moran, 1998; Liu et al., 2004b; Sidaway and Trzaska, 2005) that was also analyzed. Furthermore, a search for new literature in PubMed using the terms [Motor AND imagery AND familiarization] and [mental AND practice AND familiarization] for the period of 2010/5 to 2011/12 was done, which revealed no additional studies except the one by Schuster et al. (2011).

The theory of teaching psychology was employed for two reasons (Marzano, 1988; Klauer and Leutner, 2012a,b): (1) To ensure that the structure and the instruction mode of the MIIP would support the processing and retrieval of the collected information in patients with CNS lesions and sensorimotor impairments and (2) to increase patients' level of declarative and procedural MI knowledge. A combination of a meaningful verbal form of learning defined by Ausubel and a discovering form of learning defined by Brunner was chosen (Edelmann and Wittmann, 2012). This approach supports motivation and active engagement. For the MIIP this meant that old but unconscious experience with MI in the study patients had to be discovered and linked to new knowledge and that the program had to start with simple topics progressing to more complex tasks at the end. Based on the collected information and the clinical expertise in the neurorehabilitation center a first draft of the MIIP was developed and reviewed by two external experts of the MI field. Eventually, the final MIIP was modified based on their comments and will be described in the following section.

#### The motor imagery introduction program (MIIP)

The MIIP consists of three introduction/familiarization sessions of 30 min each. The sessions can either be provided as group or single patient intervention. For motivational (Gauthier et al., 1987) and economic reasons the group format is to be preferred. To allow individualized instructions, a maximum of four participants per group is recommended. The program is designed for patients with sensorimotor impairments such as stroke, Parkinson's disease (PD), multiple sclerosis (MS), and traumatic brain injury (TBI). To meet the cognitive demands of the program, patients are required to have a mini-mental state examination (MMSE) Score of more than 20 points and must be capable of reading and understanding German.

The program comprises MI theory (definition, type, mode, perspective, planning) and MI practice (performance, control). In each session, information is presented using power point presentations. All the three sessions have the same structure to simplify orientation. To ensure standardized sequences, detailed information for the instructors is included for each slide. Instructors have to be either physiotherapists or occupational therapists with at least 2 years of work experience with patients with sensorimotor impairments as well as profound knowledge and experience with MI. Depending on group size only a minimum of material is required: a room for group meetings with a table and chairs, a computer with a beamer, stopwatches, pencils, cups, and drinking water. An additional instructor manual provides detailed information about the goal of the program, inclusion criteria for participants, theoretical MI background, required MI knowledge of the staff, format and material used, as well as the media and preparation required for each session. Furthermore, the manual includes an overview table for each session, showing the content and the underlying theory for each slide in chronological order. A minimum of preparation time before/after each session is required.

***Session 1.*** First the study patients learn, that the goal of the MIIP is to familiarize them with the concept of MI and that the MIIP serves as a standardized pre-training before individualized MI training in combination with classical therapy starts. Study patients learn that MI is the ability to mentally simulate actions and movements; that this ability has been used in sports as a technique to improve motor performance for a long time and that over the last two decades this concept has become more and more popular in neurorehablitation. They learn that the concept is widely used in clinical routine, but still lacks sound scientific evidence. They learn how MI is believed to function in a very simplified manner. Study patients are prompted to link this theory to their own (preferably positive) experiences and a first standardized practical MI exercise is performed: lifting up a cup from the table and bringing it to the mouth and drink. Afterwards, study patients are asked to repeat this exercise until the next session. To support their MI practice, an instruction sheet describing the home exercise is provided. At the end of each session a summary is given to consolidate the new knowledge.

***Session 2.*** At the beginning there is a brief repetition of the content of the previous session. Then, study patients have the opportunity to share their experiences with their home exercises. In the theoretical part of session two, patients learn that MI in neurorehabilitation is used as an additional therapy to the classical active therapies. They also learn that the goal of MI is to increase motor performance and that MI is a possible treatment technique for all patients who have a basic ability to perform MI. They learn that MI can be performed everywhere as soon as the technique is mastered even in the absence of a therapist. They learn that the technique is not physically exhausting, but that they need to be alert and be able to concentrate to the mental representation of a given movement or action. Then the terms “modality” and “perspective” are introduced and explained with pictures and rehearsed practically. With the new knowledge, the exercise instructed in session one is repeated and the patients now have the possibility to share their own perceptions. At this point a second movement is chosen by the patient and individualized according to his situation. Each study patient receives a second instruction sheet describing his individualized exercise. Session two is concluded with a summary of the new session content.

***Session 3.*** The session starts with a repetition and sharing of experiences at the beginning and a summary of the new content at the end of the session. In the theoretical part of session three the patients learn that MI can be used throughout the day, whenever the time is right for the patient and that no special material is needed. They learn more about details of the process, such as starting- and endpoint of an imagined movement, as well as the necessity to control the mental process. Furthermore, patients learn about and experience different qualities of these mental representations and how they can be described. With the new knowledge, patients have the opportunity to mentally practice their individualized exercise. At the end of session three there is time for group reflection and a short summary of the most important components of the program: the **what**, **who**, **where**, **when** and **how** of MI. A summary of the content of each session is provided in Table 1.

**Table 1:** **Content summary of each MIIP session**.

**Nr.**	**Theory**	**Practical exercises**	**Assignment**
1	Overview of the content What MI is How MI works Since when MI is being used in NR Summary at the end of the session	Link to own experience First standardized exercise	Instruction sheet with standardized exercise
2	Repetition and sharing of experience MI goals in NR Who can benefit from MI Where and how MI can be executed Terms “perspective” and “modality” are introduced Summary at the end of the session	Repetition of standardized exercise Individualized exercise	Instruction sheet with individualized exercise
3	Repetition and sharing of experience When MI can be used Material needed Starting- and endpoint of one MI trial Necessity to control the mental process Group reflection and summary	Practice of individualized exercise

NR, neurorehabilitation; MI, motor imagery.

### Phase 2: development of the MIIP-evaluation-questionnaire (MIIP-EQ)

The objective of Phase 2 was the development of a self-administered, paper-based questionnaire in German as an instrument to evaluate the MIIP that was developed in Phase 1. Based on literature about program evaluation and questionnaire construction (Bühner, 2006; Clasen, 2010), and after a content analysis of the MIIP, a first draft of the MIIP-EQ was developed consisting of three parts: A, B, and C. Part A was designed to assess declarative knowledge, part B to assess procedural knowledge with the self-perception of the skills to perform MI, and part C to assess patient-satisfaction. For part C the “Zufriedenheit-8” questionnaire (ZUF-8), an existing questionnaire about patient satisfaction with 8 items in German served as information source (Schmidt et al., 1989). Six questions could be adopted with minor modifications and four more MIIP specific questions were added. For all the three subscales, items in form of questions were developed: part A: 16 items, part B: 10 items, part C: 15 items. For face validity, the same two external experts, who had reviewed the MIIP, also evaluated the preliminary MIIP-EQ collection of items regarding their relevance and to delete or add items if necessary. Based on the results of the external reviewing process and on own clinic expertise, the MIIP-EQ draft was modified and finalized: part A: 12 items, part B: 5 items, part C: 10 items.

In the final version, part A of the MIIP-EQ consisted of 12 multiple-choice questions. For each of the questions, four answers were given (one correct and three false). Each correct answer received a score of 1, for a wrong answer the score was 0. This resulted in a total knowledge score of 12 if all answers were correct (range 0–12). Three questions tested basic knowledge about MI, such as the meaning of MI, the goal of MI and MI processing in the brain in a simplified manner. Two questions asked about the practical execution of MI. Three questions focused on modality and perspective, and one question tested possible terms used to describe the quality of perceived MI. A further question evaluated knowledge about mental chronometry and time equivalence when performing MI compared to physical practice. The content of the individual questions is presented in an abbreviated form in Table 2. For the evaluation of the knowledge transfer process the minimal score level to be sufficient was set at eight to ten (60% to 87%) correct answers. Twelve and eleven correct answers were regarded as excellent (88% to 100%). Seven or less correct answers were regarded as insufficient (< 60%). This was in accordance with the censoring model proposed by Klauer and Leutner (2012a,b).

**Table 2:** **MIIP-EQ part A: correct answers per item for all study patients**.

**Item-Nr.**	**MIIP-EQ part A: items asked**	**Number of correct answers *n* (%)**	
	**Abbreviated answer content**	**Pretest**	**Posttest**
1	Meaning of the term MI	7 (63.6%)	8 (72.7%)
2	Meaning of the term modality	3 (27.3%)	7 (63.6%)
3	Different qualities of MI modality: kinesthetic/visual	1 (9.1%)	9 (81.8%)
4	Meaning of the term MI perspective: internal/external	1 (9.1%)	5 (45.5%)
5	Description of the quality of MI	3 (27.3%)	10 (90.9%)
6	Simplified theory about the mechanism of MI	4 (36.3%)	7 (3.6%)
7	Goal of MI	10 (90.9%)	11 (100%)
8	Correct performance of one MI trial	7 (63.6%)	9 (81.8%)
9	Different phases of MI per trial: planning, execution, controlling	6 (54.5%)	5 (45.5%)
10	MI ability after central nervous system lesion	6 (54.5%)	6 (54.5%)
11	Advantages and benefits of MI	10 (90.9%)	9 (81.8%)
12	Definition of mental chronometry	1 (9.1%)	8 (%)

MI, motor imagery; MIIP-EQ, motor imagery introduction program evaluation questionnaire.

Part B of the MIIP-EQ consisted of five questions regarding different aspects of the skill to perform MI, e.g., during therapy sessions or at home. The patients were asked to evaluate their self-perception on an ordinal ranked unipolar six point Likert scale. The anchors were “very low” at the left end and “very good” at the right end. The total score of part B was 25 points (range 0–25).

Part C of the MIIP-EQ was only part of the post assessment and evaluated the participants' satisfaction with the MIIP. The study patients could rate their satisfaction with the MIIP on an ordinal ranked unipolar six point Likert scale. The anchors were “not at all satisfied” and “very satisfied.” Originally, this part consisted of ten questions. Due to an incorrect formulation problem, one question had to be excluded during the analysis. The total score of the revised part C was therefore 45 points (range 0–45). An accept satisfaction level was set at 80% of the maximum $\left(\text{Patient satisfaction}=\frac{\text{patient score}}{\text{maximal possible score}}*100\right)$, in accordance with literature on measuring patient satisfaction (Wüthrich-Schneider, 2000).

### Phase 3: pre-evaluation of MIIP and MIIP-EQ

The objective of Phase 3 was to test the understandability of the MIIP and the MIIP-EQ in two pilot study patients. Inclusion criteria were sensorimotor impairments such as stroke, PD, MS, TBI, age older than 18 years, a MMSE score of more than 20 and the capability of reading and understanding German. Exclusion criteria were the presence of more than one of the above-mentioned diagnoses and previous experience with MI. Two inpatients met the inclusion criteria, one with MS, and one patient after stroke. After receiving oral and written information about the pre-evaluation and signing an informed consent form, they underwent the MMSE screening and were then included. In two separate single subject sessions (1.5 h per session), the study patients were introduced to MIIP and the MIIP-EQ draft. After each session, a semi-structured interview was conducted, covering the following areas: comprehensibility of the entire MIIP and MIIP-EQ, as well as comprehensibility of each MIIP slide and of MI specific terms after explanation. The answers were recorded and written notes were taken. The study patients had mainly comments regarding technical terms. They felt overwhelmed by the amount of MI specific terminology. Based on this information the drafts of the MIIP and the MIIP-EQ were revised. All technical terms, which were not absolutely necessary for patients or study participants in order to understand MI, were replaced by plain language terms.

### Phase 4: evaluation of the MIIP

The objective of the last phase was to evaluate the MIIP in a pilot trial. Our hypothesis was that MIIP would (a) increase the declarative knowledge about MI, (b) improve procedural knowledge about MI measured with the self-perception of the skill to perform MI, and (c) result in a good overall satisfaction with The MIIP. Furthermore, it was of interest whether such a program would change MI ability.

#### Study patients

The study was conducted at the rehabilitation center Rheinfelden. Between October and December 2012, inpatients as well as outpatients from the clinic were invited to participate in the study according to their diagnoses. The same eligibility criteria as in the pre-evaluation (Phase 3) were applied. After receiving oral and written information and signing the informed consent form, 12 study patients were screened for meeting inclusion criteria/eligibility. All 12 study patients were eligible and underwent pretest assessment. Information about age, gender, educational level, duration of the impairments, living situation, dominant and affected side and other therapies was recorded. One study patient did not complete the study due to a norovirus infection. His data was not included in the final data analysis. Eleven study patients underwent posttest assessment.

#### Procedure

Different parameters regarding MI were assessed before and after the MIIP. In study week 1, the study patients were screened and underwent pretest assessment. All assessments were performed by two physiotherapists, which had been trained by an MI expert of the clinic. In study week 2, the MIIP, consisting of 3 × 30 min introduction sessions (as described above in the “program development” section) was conducted. After finalization of MIIP in study week 2 or 3 all posttest assessments were performed by two physiotherapists, who had not been involved in the instruction of the MIIP. Approximately 1.5 h were used for pre-and post-assessments. Additionally, patients' comments regarding the MIIP were noted/recorded in an open unstructured form. Due to recruitment the first group consisted of 3 participants, the second of 1 participant, the third of 3, the fourth of 2 and the fifth of 3 participants (*n* = 12).

#### Outcome measures

The primary outcome and secondary outcomes and corresponding measures are described in Table 3.

**Table 3:** **Primary and secondary study endpoints and corresponding outcome measures**.

**Primary study endpoint**	**Primary outcome measure**
Change of declarative knowledge about MI	Part A of the MIIP-EQ: Knowledge score (max. score 12 points, range 0–12 points, 6-point Likert scale)
**Secondary study endpoints**	**Secondary outcome measures**
Change of procedural knowledge about MI	Part B of the MIIP-EQ: self-perception of MI performance skills (max. score 25 points, range 0–25 points, 6-point Likert scale)
Study patient satisfaction with MIIP	Part C of the MIIP-EQ (max. score 45 points, range 0–45 points, 6- point Likert scale)
Change in the MI ability	KVIQ_*vis*_-G 20 and KVIQ_*kin*_-G 20 (max. score 50 points, range 10–50 points for each subscale)
	Imaprax-G vividness total score
	(Imaprax-Software, Version 1.1) (max. score 42 points, range 6–42 points)
Change in mental chronometry ratio	Change in the time congruence between the time needed to imagine a specific movement and the time needed to actually perform the same movement, expressed as:
	$\text{ratio}=\frac{\text{I}=\text{time to imagine the movment (s)}}{\text{E}=\text{time to execute the movement (s)}}$

MIIP, motor imagery introduction program; MIIP-EQ, motor imagery introduction program evaluation questionnaire; KVIQ, kinesthetic and visual imagery questionnaire; G, German.

The MIIP-EQ is described in detail in Phase 2 “development of the MIIP-Evaluation-Questionnaire (MIIP-EQ)” mentioned above.

The KVIQ has specifically been developed for patients after stroke (Malouin et al., 2007) and was re-validated after translation into German for patients with sensorimotor impairments (Schuster et al., 2010). To assess MI ability, the KVIQ-G 20 measures both the perceived clarity and the perceived intensity of a given movement during imagination in a standardized way using a visual and a kinesthetic subscale. Patients have to imagine a set of standardized movements, involving the whole body and each side of the body. The movement is demonstrated once by the assessor. After a single execution of the movement, patients are asked to take an internal perspective, to imagine the movement and to rate the clarity and intensity of each mental picture on a 5 point-Likert-scale (clarity: 1 = “no image,” 5 = “image as clear as actually seeing it”; intensity: 1 = “no sensation,” 5 = “as intense as performing the movement”). The KVIQ exists in a short and a long version, with 10 questions (KVIQ-G 10) or 20 questions (KVIQ-G 20), respectively (maximum score: 100 points; range 20–100 points). To calculate subscale scores as well as the total score, the values of 10 specified items for each subscale (visual and kinesthetic) are summed up.

Imaprax-G is a standardized, computer and video based test to assess MI ability. It has specifically been developed for patients with apraxia after stroke (Fournier, 2000) and was re-validated after translation into German (Schuster et al., 2010). The test consists of six upper limb gestures presented in different video sequences. For each gesture, the understanding, the clarity of the mental representation of the movement, and the perspective taken are assessed. The clarity can be rated on a 7 point Likert scale, ranging from 1 (vividness worse than in all of the presented video sequences) to 7 (vividness better than in all of the presented video sequences, maximum score 42 points, range 6–42 points).

Mental chronometry is a reliable and valid instrument to measure the congruency between the time needed to imagine a specific movement and the time needed to actually perform the same movement (Malouin et al., 2008b). A high MI ability results in an almost perfect congruence and a ratio close to 1. For standardization the following movement was chosen: “grasp a cup, lift it up, and bring it to the lips,” because all the included study patients were able to perform this movement. Each study patient had three attempts. For each attempt, first, the movement was performed and then the image of the same movement was generated. With a stopwatch the assessor measured the time needed to perform the movement and the time needed to perform the imagination in seconds. Study patients indicated the beginning and the end of each imagined movement by nodding their heads.

### Statistics

For all analyses the Statistical Package for the Social Sciences (SPSS, IBM Inc.) version 20 was used. Patient characteristics and baseline data were analyzed using descriptive statistics. Differences between pre- and posttest results were compared using the following statistical tests for depended data: (1) Parametric data with a normal distribution (assessed with the Kolmogorov-Smirnov-Test) were analyzed using the paired *t*-test (Part A of the MIIP-EQ). (2) Non-parametric data (Part B and C of the MIIP-EQ, Imaprax and KVIQ) and not normally distributed data (mental chronometry) were tested using the Wilcoxon signed-rank test. The level of significance was set at *p* = 0.05.

## Results

### Demographics

The demographics of the study patients are reported in Table 4 and an overview over the raw scores is given in Table 6.

**Table 4:** **Demographics**.

**Patient number (*n* = 11)**	**Mean/*SD***
Gender (female/male)	*f* = 5
Age (years)	62.3 ± 14.2
More affected body side (right/left)	*r* = 4
Duration of impairments (months)	111 ± 142
MMSE	28.4 ± 1.5

MMSE, mini-mental state examination score.

### Primary outcome: improvement of declarative knowledge about MI after the MIIP: (MIIP-EQ part A)

The declarative knowledge score improved significantly from 5.4 ± 2.2 to 8.8 ± 2.9 (*p* = 0.010). The mean difference was 3.5 ± 3.6 (min −3, max + 10) points. Nine study patients could improve their knowledge after the MIIP. Only one patient showed a negative result (difference −3 points) and one showed no change in declarative knowledge. Four study patients achieved excellent results with 11 or 12 correct answers. Three study patients achieved sufficient results with 9 or 10 correct answers. Four study patients showed insufficient results with 7 or less correct answers. The numbers of correct answers per item for all study patients is given in Table 5. An overview on the individual study patient results in the pretest- and posttest assessment is provided in Table 6. Statistical results are provided in Table 7.

**Table 5:** **Patient satisfaction scores of MIIP part C (possible range 0–5)**.

**Item-Nr.**	**Abbreviated content**	**mean ± *SD* (*n* = 11)**	**% of maximum**
1	Satisfaction with the overall quality of the MIIP	3.8 ± 0.9	76
2	Satisfaction with the fulfillment of expectations	3.8 ± 1.0	76
3	Satisfaction with the organization of the MIIP	4.0 ± 0.5	80
4	Satisfaction with the comprehensibility of the MIIP	4.6 ± 0.7	92
5	Perceived stress during the MIIP: No stress = 5/high stress = 0	4.6 ± 0.7	92
6	Satisfaction with degree of individualization during the MIIP	4.4 ± 0.7	88
7	Satisfaction with MI ability after the MIIP	4.2 ± 1.0	84
8	Emotional well-being during the MIIP	4.2 ± 1.0	84
9	Willingness to practice with MI after the MIIP	4.2 ± 1.0	84

MIIP, motor imagery introduction program; max, maximum percentage.

**Table 6:** **Row scores of each patient**.

**Patient number (*N* = 11)**	**Pat. 1**	**Pat. 2**	**Pat. 3**	**Pat. 4**	**Pat. 5**	**Pat. 6**	**Pat. 7**	**Pat. 8**	**Pat. 9**	**Pat. 10**	**Pat. 11**
**DEMOGRAPHICS**											
Gender (female/male)	f	f	m	m	m	m	m	f	f	m	f
Age (years)	70	76	40	52	68	75	45	43	69	72	75
Diagnosis	Stroke	Stroke	MS	TBI	Stroke	Stroke	Stroke	MS	MS	PD	MS
More affected body side (right/left)	r	l	r	l	l	l	l	r	l	r	r
Duration of impairments (months)	2	4	290	1	8	63	1	192	420	60	180
MMSE	28	28	28	29	28	30	30	29	30	25	27
**MIIP-EQ QUESTIONNAIRE**											
MIIP-EQ: knowledge pretest (range 0 to 12)	5	3	4	6	2	4	9	7	7	4	8
MIIP-EQ: knowledge posttest (range 0 to 12)	6	7	12	10	12	9	12	9	11	4	5
Difference MIIP knowledge (range −12 to +12)	1	4	8	4	10	5	3	2	4	0	−3
MIIP-EQ part B pretest(range 0 to 25)	14	18	5	20	18	17	20	14	20	17	15
MIIP-EQ part B posttest (range 0 to 25)	21	16	21	18	16	19	21	21	19	16	15
Difference MIIP-EQ (range −25 to +25)	7	−2	16	−2	−2	2	1	7	−1	−1	0
MIIP-EQ: Patient satisfaction (range 0 to 45/%)	40/89	35/78	35/78	40/89	39/87	44/98	40/89	37/80	44/98	30/67	28/62
**IMAPRAX-G CLARITY PRETEST (RANGE 6–42)**	34	35	32	30	25	35	36	36	36	33	34
gesture 1: to beckon somebody (identified gesture/level of clarity/perspective used)	P/5/E	P/5/E	P/5/E	T/3/E	T/4/I	P/6/E	T/6/E	P/6/I	P/6/E	P/6/I	F/6/E
gesture 2: to cut something	F/6/E	T/6/E	T/5/I	T/4/I	T/5/I	T/6/E	T/6/I	T/6/I	T/6/I	T/6/E	T/6/E
gesture 3: to write something	P/6/E	P/6/E	T/5/E	P/6/E	P/4/I	P/6/I	T/6/I	T/6/I	T/6/I	T/6/E	P/6/E
gesture 4: to brush one's teeth	T/6/E	T/6/E	T/6/E	T/6/E	P/5/E	P/6/E	T/6/E	T/6/I	T/6/E	T/5/E	T/6/E
gesture 5: to cock a snook	T/6/E	T/6/E	T/6/E	P/5/E	T/3/E	T/5/E	T/6/E	T/6/I	T/6/E	T/6/E	T/4/E
gesture 6: to applaud somebody	T/5/E	T/6/E	T/5/E	T/6/E	T/4/I	T/6/E	T/6/I	T/6/I	T/6/E	T/5/E	T/6/E
**IMAPRAX-G POSTTEST (RANGE 6–42)**	36	35	36	35	29	34	36	36	36	29	27
gesture 1: to beckon somebody	P/6/E	P/6/E	T/6/E	T/5/E	T/5/E	P/5/E	T/6/I	T/6/I	P/6/E	T/3/I	T/4/E
gesture 2: to cut something	T/6/E	T/6/E	T/6/E	T/6/E	T/5/E	T/6/E	T/6/I	T/6/I	T/6/E	P/5/E	T/4/E
gesture 3: to write something	T/6/E	P/6/E	T/6/E	T/6/E	T/4/I	T/5/I	T/6/I	T/6/I	T/6/E	P/6/E	P/6/E
gesture 4: to brush one's teeth	T/6/E	P/6/E	T/6/E	T/6/E	T/5/E	T/6/E	T/6/I	P/6/I	P/6/E	F/5/E	P/5/E
gesture 5: to cock a snook	T/6/E	T/5/E	T/6/E	T/6/E	T/6/E	T/6/I	T/6/I	T/6/I	T/6/E	T/6/E	T/2/E
gesture 6: to applaud somebody	T/6/E	T/6/E	T/6/E	T/6/E	T/4/I	T/6/I	T/6/I	T/6/I	T/6/E	P/4/I	T/6/E
**MENTAL CHRONOMETRY PRETEST**											
Mean MI time of 3 attempts (s)	1.7	8.6	3.2	2.1	5.2	2.2	1.5	3.3	1.6	3.5	3.0
Mean movement time of 3 attempts (s)	3.7	7.5	3.5	1.4	2.8	2.1	1.2	2.4	2.1	5.6	2.9
Ratio	0.5	1.2	0.9	1.5	1.9	1.1	1.2	1.4	0.8	0.6	1.0
**MENTAL CHRONOMETRY POSTTEST**											
Mean MI time of 3 attempts (s)	2.4	10.6	4.3	1.1	4.7	5.3	2.7	2.6	3.0	3.0	2.6
Mean movement time of 3 attempts (s)	4.8	7.3	3.7	1.6	2.4	6.7	3.8	2.1	3.8	2.3	2.3
Ratio	0.5	1.4	1.2	0.7	2.0	0.8	0.7	1.3	0.8	1.3	1.2
**KVIQ-20 CLARITY TEST**											
KVIQ-20 pretest (10–50)—vis. subscale	39	29	38	39	33	42	46	10	47	31	44
KVIQ-20 posttest (10–50)—vis. subscale	39	26	38	38	31	39	47	40	42	28	39
KVIQ-20 pretest (10–50)—kin. subscale	43	39	37	31	24	22	39	15	46	32	41
KVIQ-20 posttest (10–50)—kin. subscale	37	38	36	29	27	12	36	20	36	26	30

MS, multiple sclerosis; TBI, traumatic brain injury; PD, Parkinson's disease; MMSE, mini-mental state examination score; MIIP-EQ, motor imagery introduction program evaluation questionnaire; P, partially true; F, false; T, true; E, external; I, internal; KVIQ, kinesthetic and visual imagery questionnaire.

**Table 7:** **Statistical results**.

	**Mean/*SD***		**Median (range)**	***z*-value**	***p*-value**
**MIIP-EQ QUESTIONNAIRE**					
MIIP-EQ: knowledge pretest (range 0–12)	5.4 ± 2.2	3.5 ± 3.6			*p* = 0.014
MIIP-EQ: knowledge posttest (range 0–12)	8.8 ± 2.9				
MIIP-EQ part B pretest(range 0–25)	16.2 ± 4.3		17 (5–20)	−0.721	*p* = 0.471
MIIP-EQ part B posttest (range 0–25)	18.5 ± 2.4		19 (15–21)		
MIIP-EQ: Patient satisfaction (range 0–45/%)	37.5 ± 5	83.2 ± 11.4%			
Imaprax-G clarity pretest (range 6–42)	33.27 ± 3.3		34 (25–36)	−0.341	*p* = 0.733
Imaprax-G clarity posttest (range 6–42)	33.6 ± 3.5		35 (27–36)		
**KVIQ-20 CLARITY TEST**					
KVIQ-20 pretest (10–50)—vis. subscale	36.2 ± 10.5		39 (10–47)	−1.424	*p* = 0.153
KVIQ-20 posttest (10–50)—vis. subscale	37 ± 6.2		39 (26–47)		
KVIQ-20 pretest (10–50)—kin. subscale	33.6 ± 9.7		37 (15–46)	−2.004	*p* = 0.045
KVIQ-20 posttest (10–50)—kin. subscale	29.7 ± 8.2		30 (12–38)		
MC pretest Mean MI time of 3 attempts (s)	3.3 ± 2.1	Ratio 1.1 ± 0.4	1.05 (0.47–1.85)	−0.178	*p* = 0.859
MC pretest Mean movement time: 3 attempts (s)	3.2 ± 1.9				
MC posttest Mean MI time of 3 attempts (s)	3.9 ± 2.6	Ratio 1.1 ± 0.5	1.16 (0.49–1.97)		
MC posttest Mean movement time: 3 attempts (s)	3.7 ± 1.9				

MIIP-EQ, motor imagery introduction program evaluation questionnaire; MC, mental chronometry; KVIQ, kinesthetic and visual imagery questionnaire; s, seconds.

### Secondary outcomes

#### Change of procedural knowledge about MI after the MIIP (MIIP-EQ part B)

The procedural knowledge about MI measured with the elf-evaluation of the MI performance skill (MIIP-EQ, part B) changed non-significantly from 16.2 ± 4.3 to 18.5 ± 2.4 (*z* = −0.721, *p* = 0.47). The mean increase was 2.3 ± 5.6 points (range from −2 to +16). Five study patients rated their skill to perform MI in the posttest assessment lower than in the pretest assessment (difference −2 in 3 study patients, difference −1 in 2 study patients) and one study patient showed no change. An overview of the results for each study patient is displayed in Table 6. Statistical results are provided in Table 7.

#### Study patient satisfaction with the MIIP (MIIP-EQ part C)

The mean total satisfaction score was 37.5 ± 5.2 (range 28–44) or 83.2 ± 11.4%, i.e., above the level of 80% defining good satisfaction. Interestingly, the two study patients with the lowest MMSE also showed the lowest satisfaction score with the MIIP. An overview of the results for each study patient is shown in Table 4. The mean satisfaction score per item is reported in Table 5.

#### KVIQ-G 20

There was no significant change in the visual subscale of the KVIQ-G 20 (*z* = −1.424, *p* = 0.153). The kinesthetic subscale of the KVIQ-G 20 decreased significantly (*z* = −2.004, *p* = 0.045). Results of the KVIQ-20 for each study patient are shown in Table 6. Statistical results are provided in Table 7.

#### Imaprax-G

There was no significant change in the vividness score (*z* = −0.341, *p* = 0.733). All results for each study patient are shown in Table 6 and an overview over the statistical results is reported in Table 7.

#### Mental chronometry

There was no significant change in mental chronometry (*z* = −0.178, *p* = 0.86). At pretest, mean time to perform MI was 3.3 ± 2.1 s (range 1.5–8.6 s). Mean time to physically perform the movement was 3.2 ± 1.9 s (range 1.2–7.5 s). Pretest ratio was 1.1 ± 0.4 (range 0.5–1.9).

At posttest assessment, mean time to perform MI was 3.9 ± 2.6 s (range 1.2–10.6 s). Mean time to physically perform the movement was 3.7 ± 1.9 s (range 1.6–7.3 s). Posttest ratio was 1.1 ± 0.5 (range 0.5–2). Results of each individual study patient are shown in Table 6 and an overview over the statistical results is reported in Table 7.

#### Open patient comments

In general the study patients were very interested in the program. They participated actively and contributed to a good atmosphere during the group sessions. Six of the study patients mentioned a personal benefit from the program. The perceived personal benefit concerned functional improvements, e.g., dressing, selective range of motion in four and better MI abilities in five study patients. Two of them mentioned that kinesthetic MI is difficult for them to practice, and two others mentioned that the pace of the program could have been higher.

## Discussion

Although, there is consensus in the literature that MI is a cognitively complex and challenging concept (Lotze and Halsband, 2006; Schuster et al., 2012a), familiarization has so far not been regarded as a key element of MI. A familiarization process has not been standardized or systematically evaluated. The aim of our study was to develop a standardized MIIP for patients with a CNS lesion and sensorimotor impairments, with the intention to improve their declarative and procedural knowledge about MI and to evaluate this MIIP in a pilot trial.

The whole process was structured into four phases: in phase one the MIIP and in phase two the corresponding evaluation questionnaire (MIIP-EQ) were developed. In phase three these two elements were pre-evaluated and modified and in phase four the revised versions were evaluated in a pilot patient trial.

During the development process of the MIIP was distinguished between declarative and procedural knowledge (Annett, 1996). The MIIP was designed for practical use in clinical routine. A manual for therapists was created to facilitate implementation of the whole program into clinical practice or its use in clinical studies. With only 3 × 30 min instruction time and a minimum of preparation time, overall time expenditure is well manageable. Group size can be varied between one and four patients, offering great flexibility to fit the program to different clinical situations and busy patient schedules.

The MIIP-EQ was developed to guarantee an objective MIIP evaluation. Three different aspects were regarded as important to be evaluated: (1) the success of the intended declarative and (2) procedural knowledge transfer, and (3) patient satisfaction.

The pre-evaluation phase proved to be important to detect incomprehensibilities in the MIIP and the MIIP-EQ, saving both personal and patient resources and improving quality of the data generated in the pilot trial.

In the pilot trial, we found that the MIIP significantly increased declarative MI knowledge in the majority of our study patients. This finding supports the hypothesis that the MIIP is a feasible tool to transfer declarative MI knowledge in patients with sensorimotor impairments. After the MIIP, the majority of the study patients showed a sufficient to excellent level of declarative MI knowledge (Klauer and Leutner, 2012a,b). It can be assumed that consequent implementation of such a structured instruction program would enhance the clinical benefit that patients could derive from MI, but this would need to be tested in an adequately designed clinical trial.

However, even after the MIIP, still a majority of the study patients could not link the word “perspective” to the words “external and internal,” and detecting the correct phases of one MI sequence remained difficult. This may have two reasons: Either there was not enough emphasis on this fact during the sessions or the perspectives as a construct are too complex to understand for some patients. This raises questions about what patients are doing when they are asked to take a certain perspective to perform MI either in test situations or in therapy. Since this is crucial for obtaining valid MI assessment results and for being able to practice MI in the future, more attention has to be paid to these aspects. When applying the MIIP in our clinic, we decided to put more emphasis on this by having patients describe the perspective they have taken.

In only two out of eleven patients declarative MI knowledge did not improve. Additionally, both patients also rated lowest on the absolute posttest knowledge score. Interestingly, the same two patients had the lowest MMSE scores (25 and 27 points, respectively) in the screening examination. The observation that knowledge gain in the context of a complex concept such as MI requires a relatively high level of cognitive abilities should be taken into account for future research on MI and the clinical applications of MI.

The hypothesis that the level of procedural knowledge would significantly increase after the MIIP was not supported by our results. The self-perception of the skill to perform MI showed no significant improvement after the MIIP. Five patients even rated their skill to perform MI in the posttest assessment lower than in the pretest assessment. A possible explanation for this could be that with better declarative knowledge of the MI concept after the MIIP, some patients might have rated their skill to perform MI more accurately than at the beginning (where they might have overestimated their true skill level). This could have disguised a possible beneficial effect of the MIIP (Schuster et al., 2010). Again, the two study patients with the lowest MMSE scores at screening also showed the lowest scores in the self-perception of the skill to perform MI. This may indicate that a low level of declarative knowledge negatively influences procedural knowledge, although the results do not consistently show this. Based on these observations it can be proposed that MMSE performance should be considered to allocate patients to different MI group levels. This could help to meet the different demands of patients with varying cognitive abilities. In this respect, our results are in accordance with the findings of other investigators, who had observed that even after MI training barriers remained that compromised the motivation of the patients to practice MI (Bovend'eerdt et al., 2010). To promote the concept of MI, factors that negatively influence self-perception of the skill to perform MI or the motivation of the patient to practice MI, such as limited cognitive capacity, should be systematically evaluated in future studies. Overall, study patients were satisfied with the MIIP. However, the two study patients with the lowest MMSE scores also showed the lowest satisfaction scores. It seems that patient satisfaction can only be influenced to a certain level by external parameters. It can be assumed that increasing discrepancy between cognitive ability of the patient and the demand of the program will raise the level of frustration and negatively impact satisfaction scores.

All measured parameters regarding MI ability did not change significantly after the MIIP, supporting their retest reliability. However, the total score of the KVIQ kinesthetic subscale did change significantly toward a reduction in MI vividness. The significant difference is in accordance with findings of another study (Schuster et al., 2010) and supports the fact that with improved understanding of the concept, self-rating becomes more accurate.

Of special interest are the two patients, who scored lowest on the posttest knowledge score, in the self-perception of the skill to perform MI and the patient satisfaction, but showed relatively high scores in the MI ability questionnaires. It remains unclear, how they rated for example their kinesthetic sensations, without having a cognitive construct of the underlying theory and without a basic understanding of the terms used. This supports the idea that MI ability has to be assessed with a number of tools to get a more comprehensive idea of the patient's true MI ability.

### Limitations

A limitation of the MIIP and the MIIP-EQ development process was, that it did not try to reach consensus among the MIIP developer and the reviewers. However, this would have required a lengthy Delphi procedure, which was not justifiable considering the limited resources and the overall impact of the study question. Since there is no generally accepted standard MIIP that could have been used as gold standard, the authors did not use a control group in our pilot trial. As there is no plausible reason why patients should have gained knowledge just by chance, the increase in knowledge seen in this study can be attributed to the MIIP. A limitation of the MIIP-EQ was that it was solely developed for this study and was therefore not tested for its psychometric validity. The validity of the pilot trial is limited by the small sample size and the great heterogeneity between study patients regarding age, diagnoses, and onset of the impairment. This makes statistical interpretations difficult. However, it should be realized, that in clinical practice such heterogeneity is encountered and therefore the included study patients represent a “real world sample” thus strengthening external validity of the study findings. A further limitation was the lack of a follow up examination to determine for how long the gained knowledge can be maintained, and whether this gain in knowledge translates into a clinical benefit such as improved outcome for the patients.

## Conclusions

It can be concluded that the developed MIIP is a feasible intervention to introduce and familiarize patients with the included diagnoses and with sufficient cognitive abilities to the concept of MI. With the MIIP there is now an instrument available that is easy to use and might help to introduce patients to the MI concept and to prepare them for MI training. This may improve long-term motivation and adherence.

So far there is no validated assessment tool available that is easy to handle and allows to objectively measuring mental capacity and cognitive abilities that are required for successful learning of MI. The clinical implication is that patients need to be observed very closely during their initial phase using MI. Upon this clinical possibility it has to be decided whether the patient is able to learn and perform MI with a potential benefit. This is in accordance with the statements of different other authors (Braun et al., 2008; Bovend'eerdt et al., 2012).

For the future, the possibility that low MMSE scores negatively affect the familiarization process should be evaluated in more detail. It should be analyzed what component of the different cognitive abilities, such as perception, attention, memory, motor, language, visual/spatial, execution, interferes most with a successful acquisition of MI ability. So far required MMSE scores in published MI trials showed a wide range going for example from 15 to 24 (Crosbie et al., 2004; Malouin and Richards, 2010; Schuster et al., 2012a). It might be assumed that higher cognitive abilities than previously thought are required to allow acquisition of the basic declarative and procedural MI knowledge. Furthermore, the correlation between a good introduction to MI and long-term benefits in terms of knowledge, motivation, and functional outcomes should be investigated in a randomized controlled trial.

### Conflict of interest statement

The authors declare that the research was conducted in the absence of any commercial or financial relationships that could be construed as a potential conflict of interest.
